# Inhibition of STAT3 enhances sensitivity to tamoxifen in tamoxifen-resistant breast cancer cells

**DOI:** 10.1186/s12885-021-08641-7

**Published:** 2021-08-18

**Authors:** Seo Yun Moon, Heejin Lee, Seoree Kim, Ji Hyung Hong, Sang Hoon Chun, Hee Yeon Lee, Keunsoo Kang, Ho Shik Kim, Hye Sung Won, Yoon Ho Ko

**Affiliations:** 1 Department of Biomedicine & Health Sciences, College of Medicine, The Catholic University of Korea, Seoul, Republic of Korea; 2 Cancer Research Institute, College of Medicine, The Catholic University of Korea, Seoul, Republic of Korea; 3 Division of Medical Oncology, Department of Internal Medicine, College of Medicine, The Catholic University of Korea, Seoul, Republic of Korea; 4 Department of Microbiology, College of Natural Sciences, Dankook University, Cheonan, Republic of Korea; 5 Department of Biochemistry, College of Medicine, The Catholic University of Korea, Seoul, Republic of Korea; 6 Department of Internal Medicine, Uijeongbu St. Mary’s Hospital, College of Medicine, The Catholic University of Korea, 271 Cheonbo-Ro, Uijeongbu-si, Gyeonggi-do, 11765 Republic of Korea; 7 Department of Internal Medicine, Eunpyeong St. Mary’s Hospital, College of Medicine, The Catholic University of Korea, 1021 Tongil-Ro, Eunpyeong-gu, Seoul, 03312 Republic of Korea

**Keywords:** Breast cancer, Endocrine resistance, Src, Epidermal growth factor receptor, Signal transducer and activator of transcription protein

## Abstract

**Background:**

The mechanisms of endocrine resistance are complex, and deregulation of several oncogenic signalling pathways has been proposed. We aimed to investigate the role of the EGFR and Src-mediated STAT3 signalling pathway in tamoxifen-resistant breast cancer cells.

**Methods:**

The ER-positive luminal breast cancer cell lines, MCF-7 and T47D, were used. We have established an MCF-7-derived tamoxifen-resistant cell line (TamR) by long-term culture of MCF-7 cells with 4-hydroxytamoxifen. Cell viability was determined using an MTT assay, and protein expression levels were determined using western blot. Cell cycle and annexin V staining were analysed using flow cytometry.

**Results:**

TamR cells showed decreased expression of estrogen receptor and increased expression of EGFR. TamR cells showed an acceleration of the G1 to S phase transition. The protein expression levels of phosphorylated Src, EGFR (Y845), and STAT3 was increased in TamR cells, while phosphorylated Akt was decreased. The expression of p-STAT3 was enhanced according to exposure time of tamoxifen in T47D cells, suggesting that activation of STAT3 can cause tamoxifen resistance in ER-positive breast cancer cells. Both dasatinib (Src inhibitor) and stattic (STAT3 inhibitor) inhibited cell proliferation and induced apoptosis in TamR cells. However, stattic showed a much stronger effect than dasatinib. Knockdown of STAT3 expression by siRNA had no effect on sensitivity to tamoxifen in MCF-7 cells, while that enhanced sensitivity to tamoxifen in TamR cells. There was not a significant synergistic effect of dasatinib and stattic on cell survival. TamR cells have low nuclear p21(Cip1) expression compared to MCF-7 cells and inhibition of STAT3 increased the expression of nuclear p21(Cip1) in TamR cells.

**Conclusions:**

The EGFR and Src-mediated STAT3 signalling pathway is activated in TamR cells, and inhibition of STAT3 may be a potential target in tamoxifen-resistant breast cancer. An increase in nuclear p21(Cip1) may be a key step in STAT3 inhibitor-induced cell death in TamR cells.

**Supplementary Information:**

The online version contains supplementary material available at 10.1186/s12885-021-08641-7.

## Background

Breast cancer, a life-threatening disease that is among the most common cancers in women, is classified into subtypes according to the expression of estrogen receptor (ER), progesterone receptor (PR), and human epidermal growth factor receptor 2 (HER2). Among these subtypes, hormone receptor (HR)-positive breast cancer, referring to ER- and/or PR-positive tumours, accounts for 70% of all breast cancers, and endocrine therapy such as tamoxifen or aromatase inhibitors is an important therapeutic option in HR-positive breast cancer [1]. However, some patients who initially respond to endocrine therapy eventually experience disease progression, so that this endocrine resistance remains a challenge to be solved. Activation of other oncogenic signalling pathways has been considered as one of the mechanisms of endocrine resistance [1, 2]. Therefore, inhibition of the pathways that are activated in endocrine therapy-resistant breast cancer could overcome drug resistance and induce cell death.

Src is a membrane-associated non-receptor tyrosine kinase involved in several intracellular signalling pathways. Epidermal growth factor receptor (EGFR) is a transmembrane receptor containing an extracellular ligand-binding domain and an intracellular kinase domain. In previous studies, Src has been shown to phosphorylate a specific site of tyrosine 845 (Y845) in EGFR [3]. Y845 phosphorylation of EGFR plays an important role in cellular functions, including cell cycle progression and survival. The signal transducer and activator of transcription protein (STAT) is a known mediator of the Y845 phosphorylation of EGFR and Src signalling pathway [4]. Activated via phosphorylation, STAT leads to dimerization followed by translocation to the nucleus, and then the dimer binds to its DNA binding domains, thus regulating of gene transcription [5]. Previous studies have reported that the EGFR and Src-mediated STAT3 signalling pathways can contribute to tumour aggressiveness and treatment resistance [3, 4, 6]. In this study, we investigated the roles of the EGFR and Src-mediated STAT3 signalling pathway in tamoxifen-resistant breast cancer (TamR) cells. We examined whether inhibition of Src or STAT3 can induce apoptosis in the resistant cells and thus contribute to overcoming endocrine resistance. We found that the EGFR and Src-mediated STAT3 signalling pathway was significantly activated in TamR cells. Dasatinib reduced Src expression in TamR cells, but not STAT3 expression, which was associated with modest cell death. Stattic reduced the expression levels of STAT3 and downstream pro-survival proteins, which markedly induced apoptosis in the resistant cells. In addition, we found that inhibition of STAT3 increased the expression of nuclear p21(Cip1) in TamR cells. These findings suggest a potential role of the EGFR and Src-mediated STAT3 signalling pathway in maintaining cell survival in tamoxifen-resistant breast cancer and inhibition of STAT3 may be a more effective treatment strategy for tamoxifen-resistant breast cancer.

## Methods

### Cell culture and establishment of tamoxifen-resistant breast cancer cell lines

The ER-positive luminal breast cancer cell lines, MCF-7 and T47D, were obtained from the American Type Culture Collection and Korean Cell Line Bank, respectively. MCF-7 cells were maintained in a monolayer culture in phenol-red-free MEM (Gibco 51200–038) medium with 10% fetal bovine serum (FBS), 2 mM L-glutamine and 1% penicillin/streptomycin. T47D cells were maintained in a monolayer culture in phenol-red-free RPMI 1640 (Welgene LM011–02) medium containing 10% FBS, 1% penicillin/streptomycin. MCF-7-derived TamR cells were established by long-term culture of MCF-7 cells with 4-hydroxytamoxifen (4-OH-TAM, Selleckchem, Houston, TX, USA) in estrogen-free medium as previously described [7].

### Reagents and antibodies

4-OH-TAM was dissolved in DMSO and stored as 10 and 100 mM stock solution at − 20 °C for in vitro use. Dasatinib, a small-molecule inhibitor of Src and Abl tyrosine kinases, was obtained from Cayman Chemical Company (Ann Arbor, MI, USA). Stattic, a small-molecule inhibitor of STAT3, was obtained from Sigma-Aldrich (St. Louis, MO, USA). Dasatinib and stattic were dissolved in DMSO at 20 mM and 30 mM for in vitro use. Antibodies for ERα (sc-71064), cyclin E (sc-247), glycogen synthase kinase 3β (GSK3β; sc-81462), and phospho-GSK3β (Ser9; sc-81495), GAPDH (sc-32233) were supplied by Santa Cruz Biotechnology (Santa Cruz, CA, USA). Antibodies for cell cycle regulation antibody sampler kit (cyclin D1, cyclin D3, cyclin-dependent kinase (CDK) 2, CDK4, CDK6, and p21(Cip1); #9932), retinoblastoma (Rb; #9309), phospho-Rb (Ser807/811; #9308), Akt (#4691), phospho-Akt (Ser473; #9271), mitogen-induced p70 ribosomal protein S6 Kinase (p70S6K; #9202), phospho-P70S6K (Thr389; #9205), Src (#2123), phospho-Src (Tyr416; #6943), phospho-STAT3 (Tyr705; #9145), phospho-STAT5 (Tyr694; #9359), STAT5 (#25656), EGFR (#2232), phospho-EGFR (Tyr845; #2231), extracellular signal-regulated kinases 1 and 2 (Erk1/2; #9102), phospho-Erk1/2 (Thr202/Tyr204; #9101), survivin (#2808), and Lamin B1 (#1343) were obtained from Cell Signaling Technology (Beverly, MA, USA). Antibody for cleaved poly (ADP-ribose) polymerase (PARP; ab32064) was purchased from Abcam (Cambridge, MA, USA). Antibody for STAT3 (GTX110587) was purchased from GeneTex (Irvine, TX, USA).

### Cell viability assay

Cell viability was evaluated using a Cell Counting Kit-8 (CCK-8, Dojindo Laboratories, Kumamoto, Japan). 4-OH-TAM was withdrawn from the culture medium 3 days before experiments using TamR cells. To evaluate the dose-dependent effect of 4-OH-TAM, MCF-7 and TamR cells were seeded into 96-well plates at a density of 3 × 10^3^ cells/well and then incubated for 24 h in growth medium containing 10% FBS, at 37 °C in 5% CO_2_. Cells were treated with 4-OH-TAM (0, 3, 6, and 9 μM) for 72 h, and then 10 μL of CCK-8 was added to each well. After 2–3 h of incubation, the plates were read at 450 nm. A cell viability assay after treatment with 4-OH-TAM with and without inhibitors was assessed in the same way. In the case of treatment with inhibitors, cells were pre-incubated with 7 μM stattic (10% FBS) for 4 h and 2 μM dasatinib (2% FBS) for 24 h. Next, cells were treated with 9 μM 4-OH-TAM (10% FBS) for 72 h. To examine the combined effects of dasatinib and stattic, cells were first pre-incubated with 2 μM dasatinib (2% FBS) for 24 h, followed by 7 μM stattic (10% FBS) for 4 h, after which cells in the experimental and control groups were treated with 9 μM 4-OH-TAM (10% FBS) for 72 h. Cell viability (%) was calculated relative to untreated viable cells. Experiments were performed in triplicate and the average of the control group was set at 100%.

### Annexin V staining for apoptosis

Apoptosis assay was performed with FITC Annexin V Apoptosis Detection Kit I (RUO) (BD Bioscience, San Jose, CA, USA) according to the manufacturer’s instructions. In brief, 1 × 10^6^ TamR cells were seeded into 60 mm dishes and incubated overnight. Then, cells were treated with 7 μM stattic for 4 h and 2 μM dasatinib for 24 h, after which cells were treated with 9 μM 4-OH-TAM for 24 h. Cells were trypsinized and pelleted by centrifugation. Pelleted cells were washed twice with PBS and then resuspended in 1 × binding buffer. The samples were incubated with 5 μL annexin V-FITC and 5 μL propidium iodide (PI) (Invitrogen, Carlsbad, CA, USA) for 15 min at room temperature (25 °C) in the dark. The apoptosis assay was analysed using flow cytometry with a FACSCanto II Flow Cytometer with BD FACSDiva™ software (Becton Dickinson, Franklin Lakes, NJ, USA) using untreated cells as the negative control for gating. The data were compensated and analysed using FlowJo software (version 10, Tree Star, Ashland, OR, USA).

### Cell cycle analysis

The cells were trypsinized and pelleted by centrifugation at 1200 rpm for 5 min and fixed with 70% ethanol overnight at − 20 °C. The cells were stained with 10 mg/mL PI solution (Invitrogen) containing 100 mg/mL RNase A in PBS for 30 min at room temperature. Cell cycle analysis was performed with a FACSCanto II Flow Cytometer with BD FACSDiva™ software, and the proportions of cells in different stages of cell cycle were determined by FlowJo software (sub-diploid, G0/G1, S, and G2/M phases).

### Colony-forming assay

Cells were seeded into 6-well plates at a density of 1 × 10^3^ cells/well and then incubated overnight in growth medium containing 10% FBS, at 37 °C in 5% CO_2_. After pre-incubation with 7 μM stattic (10% FBS) for 4 h and 2 μM dasatinib (2% FBS) for 24 h, cells were treated with 3 μM 4-OH-TAM (10% FBS) for 24 h and kept to form colony for 18 days. Once colonies were formed, cells were washed and fixed with 4% paraformaldehyde (Biosesang, Seongnam, Korea) for 15 min and stained with 0.05% crystal violet (Sigma-Aldrich) for 30 min at room temperature. Colonies were washed with PBS and photographed. Colonies were counted directly on the plate and calculated relative to untreated cells. Control group was set at 100%.

### Western blot analysis

The cells were washed with PBS and lysed in RIPA protein extraction lysis buffer (Elpis Biotech, Lexington, MA, USA) containing protease (Sigma-Aldrich) and phosphatase inhibitor cocktails (Sigma-Aldrich). Total protein concentrations were determined using the Bradford assay (Bio-Rad, Hercules, CA, USA) with a spectrophotometer at 595 nm. A total of 40 μg protein per lane was loaded and separated using 8–13% sodium dodecyl sulphate-polyacrylamide gel electrophoresis (SDS-PAGE) and then transferred to polyvinylidene difluoride membrane. The membranes were blocked using 5% skim milk or 3% bovine serum albumin in Tris-buffered saline and Tween 20 (TBST) at room temperature for 30 min, then incubated with the primary antibodies at 4 °C overnight. Blots were washed with TBST and then incubated for 1 h with the secondary antibodies. Chemiluminescent signals were visualized using NEW Clarity ECL substrate (GE Healthcare, Chicago, IL, USA).

### Small interfering RNA (siRNA) reverse transfections

STAT3 was knocked down by reverse transfection with siSTAT3 (5′–CAGCCTCTCTGCAGAATTCAA–3′; Genolution) RNAs, respectively. Negative control siRNA (5′–AATTCTCCGAACGTGTCACGT–3′; Genolution) was used as a control. siRNA reverse transfection was performed using the Lipofectamine RNAiMAX Transfection Reagent (Invitrogen), according to the manufacturer’s protocol.

### Nuclear and cytoplasmic fractionation

TamR cells were treated with inhibitors and 4-OH-TAM. Following treatment, the total cell extraction was lysed as described above. Cytoplasm and nuclear extraction were prepared using an NE-PER™ Nuclear and Cytoplasmic Extraction Reagents kit (Pierce, Rockford, IL, USA) according to the manufacturer’s instruction. Cells were washed twice with cold PBS and centrifuged at 500×*g* for 5 min to remove the supernatant, leaving the cell pellet as dry as possible. Dried cell pellets were suspended in cytoplasmic extraction reagent I (CER I) by vortexing. The suspension was incubated on ice for 10 min and cytoplasmic extraction reagent II (CER II) was added. The cytoplasmic extract was isolated by centrifugation at 16,000×*g* for 5 min. The supernatant fraction was transferred to a pre-chilled tube. The insoluble (pellet) fraction, containing nuclei, was isolated by centrifugation at 16,000×*g* for 10 min after which it was resuspended in nuclear extraction reagent. The supernatant fraction, containing the nuclear extract, was transferred to a pre-chilled tube and used for subsequent experiments.

### Data acquisition and RNA-seq data analysis

To understand tamoxifen-resistant breast cancer further, an available RNA-seq dataset (GSE111151) [8] was downloaded from the gene expression omnibus (GEO) database [9]. It used a dataset of 7 different tamoxifen-resistant breast cancer cell lines. Raw sequenced reads in the FASTQ format were downloaded and trimmed using Trim Galore (http://www.bioinformatics.babraham.ac.uk/projects/trim_galore/). STAR [10] was used to align the trimmed reads to a human reference genome (hg19 genome assembly) with default parameters. StringTie [11] was used to quantify the abundance of transcripts with mapped reads by means of transcripts per million (TPM). Gene set enrichment analysis (GSEA) [12] was conducted using the GSEAP reranked tool in the GSEA application (version 4.0) with log2 of fold-change (treatment/control) values. The biological process (BP), molecular function (MF), cellular component (CC), and KEGG pathways gene set modules were used for analyses.

### Statistical analysis

The data are presented as mean ± standard deviation (SD). Between-groups comparisons were made using an unpaired t-test with GraphPad Prism (GraphPad Software, Inc., San Diego, CA, USA). *P* < 0.05 was considered statistically significant.

## Results

### TamR cells showed G1/S cell cycle progression and EGFR overexpression

**Fig. 1:**
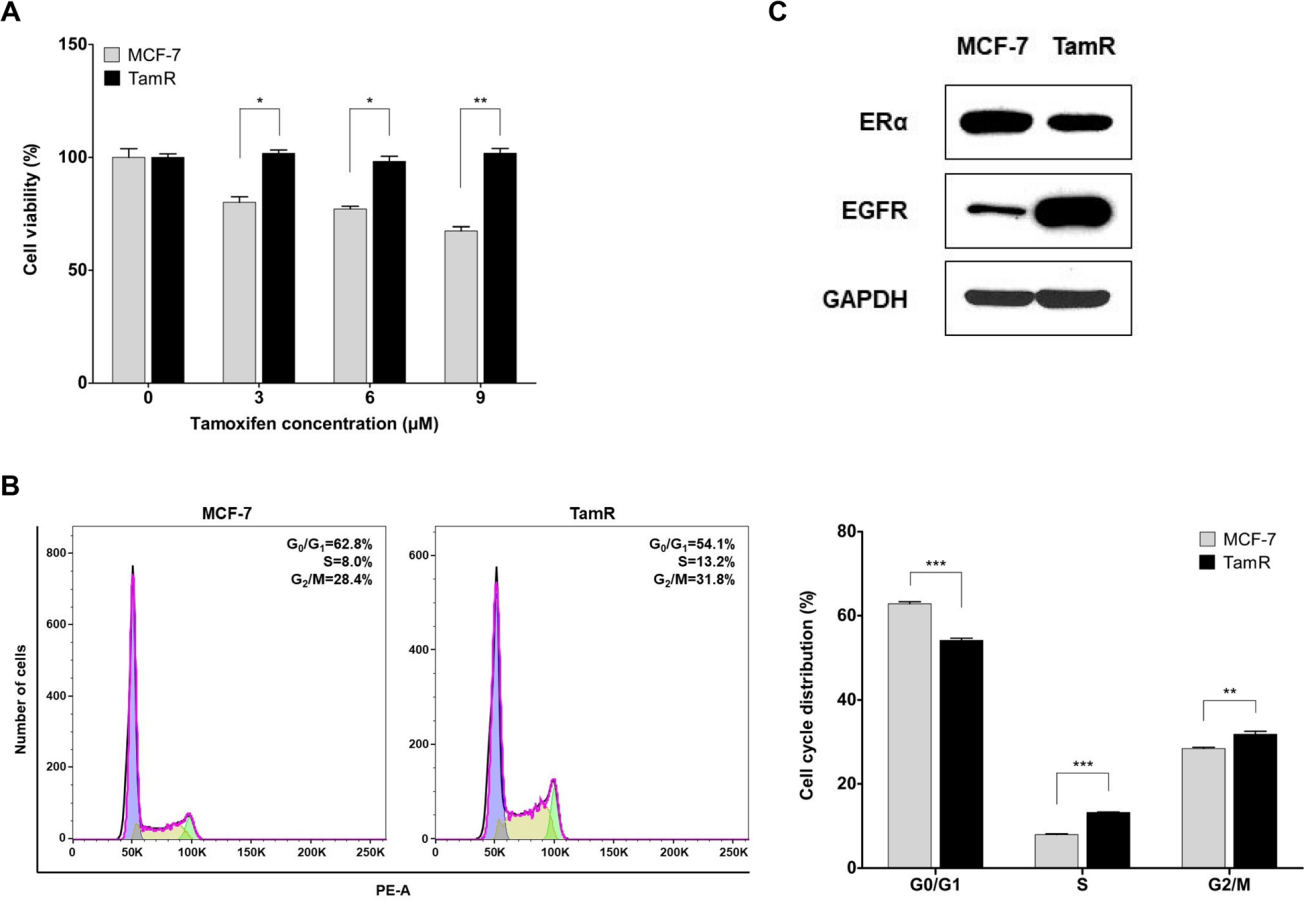
TamR cells have G1/S cell cycle progression and EGFR overexpression. **a** MCF-7 and TamR cells were treated with each concentrations of tamoxifen (4-OH-TAM) for 72 h and cell viability was measured using a CCK-8 kit. **b** Flow cytometric analysis of the cell cycle was performed in MCF-7 and TamR cells. TamR cells showed the increased G1 to S phase transition. **c** The protein expression levels of ERα and EGFR were assessed by immunoblotting in MCF-7 and TamR cells. GAPDH was used as the loading control. The cropped blots are used in the figure. The membranes were cut prior to exposure so that only the portion of gel containing the desired bands would be visualized. Data are expressed as mean ± SD, and all experiments were performed in triplicate, independently. *P* values calculated using an unpaired t-test. *p < 0.05, **p < 0.01, ***p < 0.001

To confirm the resistance to tamoxifen in TamR cells, we performed a cell viability assay after treatment with various concentrations of 4-OH-TAM in MCF-7 and TamR cells. Cell viability was significantly decreased in MCF-7 cells in a dose-dependent manner, whereas there was no change in cell viability of TamR (*p* = 0.027, 0.030, and 0.002 for 3, 6, and 9 μM, respectively) (Fig. 1a). This result indicates that TamR cells acquired resistance to tamoxifen. The cell cycle of MCF-7 and TamR cells was analysed using flow cytometry. We found alterations in the proportion of cells in G0/G1 and S phase between MCF-7 and TamR cells. TamR cells had a cell cycle shift from G0/G1 to S phase compared with MCF-7 cells, indicating that G1/S cell cycle progression was increased in TamR cells (Fig. 1b). To investigate changes in signalling pathways between MCF-7 and TamR cells, the expression levels of signalling pathway proteins were evaluated using western blot analysis. TamR cells showed low ERα and high EGFR protein expression level, while MCF-7 had high ERα and low EGFR protein expression level (Fig. 1c). This result showed that the EGFR signalling pathway was activated in the process of acquiring resistance to tamoxifen.

### EGFR and Src-mediated STAT3 signalling pathway is activated in TamR cells, but not the PI3K/Akt and MAPK/Erk signalling pathway

**Fig. 2:**
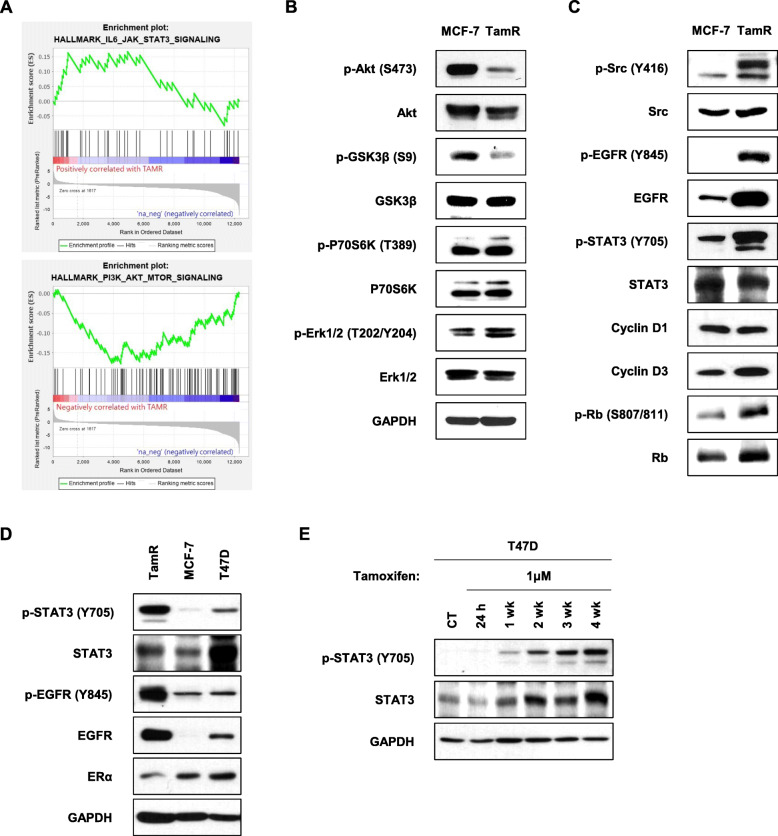
EGFR and Src-mediated STAT3 signalling pathway is activated in TamR cells. **a** GSEA enrichment plots showed enrichment of the interleukin-6/Janus Kinase (JAK)/STAT3 and PI3K/Akt signalling pathways in tamoxifen-resistant breast cancer. The red horizontal bar terminating in blue indicates a shift from positively correlated genes (red) to negatively correlated genes (blue). **b** The protein expression levels of PI3k/Akt and MAPK/Erk signalling pathways were determined using immunoblotting. **c** MCF-7 and TamR cells were analysed using western blot with the indicated antibodies to determine the expression level of the EGFR and Src-mediated STAT3 signalling pathway. **d** The baseline levels of ERα, EGFR and STAT3 protein expression were determined by immunoblotting in TamR, MCF-7 and T47D cells. **e** T47D cells were incubated with tamoxifen for 24 h, 1 week, 2 weeks, 3 weeks, and 4 weeks. STAT3 protein expression levels were determined by immunoblotting. The cropped blots are used in the figure. The membranes were cut prior to exposure so that only the portion of gel containing the desired bands would be visualized. GAPDH was used as the loading control

Overexpression of EGFR is associated with several signalling cascades involved in cell proliferation and survival. These pathways include the phosphoinositide 3-kinase (PI3K)/Akt, mitogen-activated protein kinase (MAPK), STAT3, and Src family kinases. Therefore, we investigated the changes in these signalling pathways in TamR cells. GSEA showed that genes associated with the interleukin-6/Janus Kinase (JAK)/STAT3 signalling pathway were increased in TamR cells compared with MCF-7 cells, while genes associated with the PI3K/Akt signalling pathway were also decreased in TamR cells (Fig. 2a). Activation of PI3K results in the sequential phosphorylation of Akt serine/threonine kinase and GSK3β. We found a significant decrease in both phosphorylated Akt (p-Akt) and GSK3β (p-GSK3β) protein expression level in TamR cells compared with MCF-7 cells (Fig. 2b). With respect to the MAPK/Erk signalling pathway, there was no significant change in the expression level of phosphorylated Erk (p-Erk) between TamR and MCF-7 cells (Fig. 2b). On the other hand, the expression level of phosphorylated Src (p-Src), EGFR (p-EGFR), and STAT3 (p-STAT3) was significantly increased in TamR cells compared with MCF-7 cells (Fig. 2c). Phosphorylated STAT5 (p-STAT5) was not detectable in either TamR or MCF-7 cells, and there was no difference in the expression of STAT5 between the two cell lines (Figure S1). This suggested that the EGFR and Src-mediated STAT3 signalling pathway was activated in TamR cells. We examined the expression of cell cycle regulatory molecules in both cell lines. Expression of cyclin D3 and phosphorylated Rb (p-Rb) was increased in TamR cells compared with MCF-7 cells (Fig. 2c). There were no differences in the expression of cyclin E, CDK2, CDK4, and CDK6 between the two cell lines (Figure S1). To validate this observation, we evaluated sequential changes of STAT3 activation in T47D cells exposed to tamoxifen. These two cell lines, MCF-7 and T47D, had similar phenotypes with strong expression of ER and weak expression of p-EGFR and p-STAT3 (Fig. 2d). We found that the expression of p-STAT3 was enhanced according to exposure time of tamoxifen in T47D cells, suggesting the association between STAT3 activation and tamoxifen resistance in HR-positive breast cancer cells (Fig. 2e).

### Inhibition of Src inhibits cell proliferation and induces apoptosis in TamR cells

**Fig. 3:**
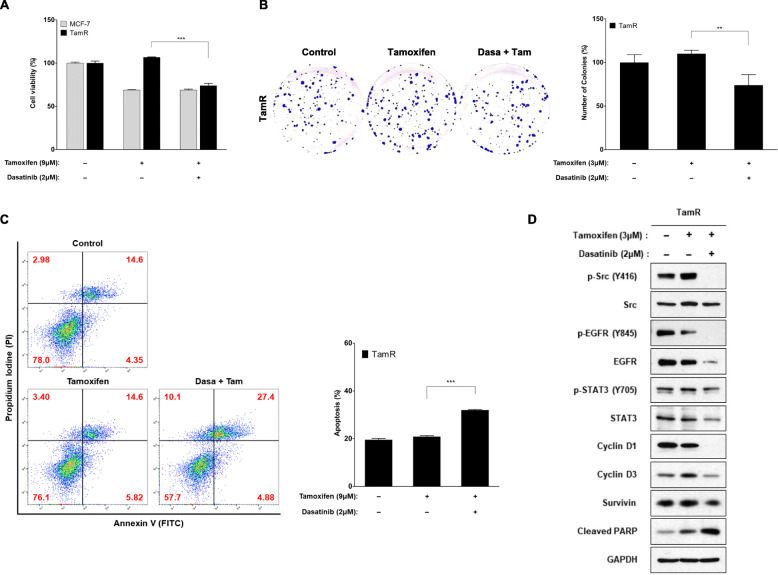
Inhibition of Src shows a modest inhibitory effect on TamR cell survival. Cells were pre-incubated with 2 μM dasatinib for 24 h and then treated with 9 μM tamoxifen for 72 h. **a** Cell viability was measured using a CCK-8 kit. **b** For colony formation, cells were kept for 18 days and then stained with crystal violet and photographed. **c** After 24 h of pre-incubation with the indicated concentration of dasatinib, cells were treated with tamoxifen for 24 h. Flow cytometric assay using annexin V-FITC and PI showed that dasatinib increased the total number of apoptotic cells. Histogram reported a summary of the apoptotic index. Annexin V (+) / PI (+) and annexin V (+) / PI (−): early and late apoptotic cells, annexin V (−) / PI (+): necrotic fraction, annexin V (−) / PI (−): live cells. **d** Lysates were immunoblotted for phospho-Src (p-Src), Src, phospho-EGFR (p-EGFR), EGFR, phospho-STAT3 (p-STAT3), STAT3, cyclin D1, cyclin D3 and cleaved PARP. The cropped blots are used in the figure. The membranes were cut prior to exposure so that only the portion of gel containing the desired bands would be visualized. GAPDH was used as the loading control. Data are expressed as mean ± SD and all experiments were performed in triplicate, independently. *P* values calculated using an unpaired t-test. *p < 0.05, **p < 0.01, ***p < 0.001

To assess the functional significance of Src expression in TamR cells, dasatinib was used to inhibit Src expression. Drug concentration and treatment time were determined by measuring concentration- and time-dependent cell viability and suppression of the target protein in MCF-7 and TamR cells. Dasatinib had only modest effects on cell proliferation, colony formation, and apoptosis in TamR cells. Cell viability assay showed that TamR cells were reduced after treatment with dasatinib by 74% compared with control or tamoxifen alone (*p* = 0.0002 and 0.0001, respectively) (Fig. 3a). Dasatinib slightly inhibited the colony formation ability of TamR cells compared with tamoxifen alone (74% vs. 110%, *p* = 0.009) (Fig. 3b). Annexin V-PI staining indicated that inhibition of Src in TamR cells increased the total number of dead cells from 19.0% (control) to 32.3% (dasatinib) (*p* = 0.0001) (Fig. 3c). Dasatinib increased apoptotic marker protein, cleaved PARP expression compared with control or tamoxifen alone, indicating that inhibition of Src can enhance the apoptosis of TamR cells (Fig. 3d). Dasatinib significantly decreased the expression level of p-Src and p-EGFR in TamR cells, but it did not affect the expression of p-STAT3 (Fig. 3d). In the expression of downstream effector molecules, dasatinib inhibited the expression of cyclin D1, but had no effect, or slightly decreased, expression of cyclin D3 and survivin (Fig. 3d).

### STAT3 inhibitor is more potent than Src inhibitor in inhibiting the growth of TamR cells

**Fig. 4:**
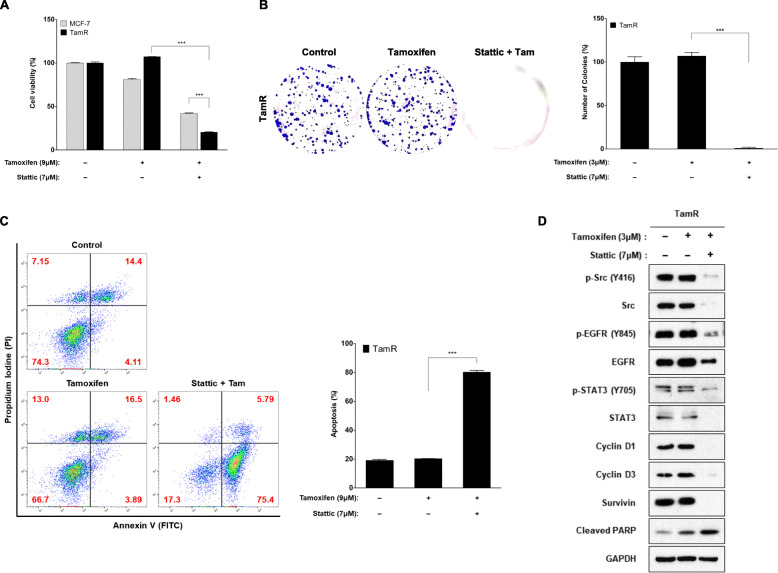
Inhibition of STAT3 shows a potent inhibitory effect on TamR cell survival. Cells were pre-incubated with 7 μM stattic for 4 h and then treated with 9 μM tamoxifen for 72 h. **a** Cell viability was measured using a CCK-8 kit. **b** For colony formation, cells were kept for 18 days and then stained with crystal violet and photographed. **c** After 4 h pre-incubation with the indicated concentration of stattic, cells were treated with tamoxifen for 24 h. Flow cytometric assay using annexin V-FITC and PI showed that stattic increased the total number of apoptotic cells. Histogram reported a summary of the apoptotic index. Annexin V (+) / PI (+) and annexin V (+) / PI (−): early and late apoptotic cells, annexin V (−) / PI (+): necrotic fraction, annexin V (−) / PI (−): live cells. **d** Lysates were immunoblotted for phospho-Src (p-Src), Src, phospho-EGFR (p-EGFR), EGFR, phospho-STAT3 (p-STAT3), STAT3, cyclin D1, cyclin D3 and cleaved PARP. The cropped blots are used in the figure. The membranes were cut prior to exposure so that only the portion of gel containing the desired bands would be visualized. GAPDH was used as the loading control. Data are expressed as mean ± SD and all experiments were performed in triplicate, independently. *P* values calculated using an unpaired t-test. *p < 0.05, **p < 0.01, ***p < 0.001

**Fig. 5:**
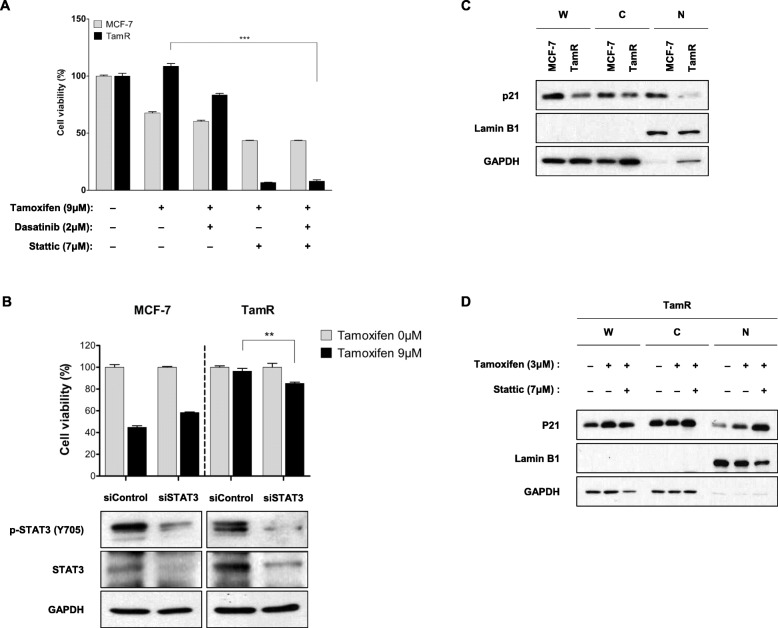
Inhibition of STAT3 induces increased expression of nuclear p21. **a** Cells were pre-incubated with the indicated concentration of stattic and dasatinib, and then treated with 9 μM tamoxifen for 72 h. Cell viability was measured using a CCK-8 kit. **b** MCF-7 cells and TamR cells were transfected with siControl and siSTAT3 (100 nmol/L). Thereafter, cells were treated with 9 μM tamoxifen for 72 h. Knockdown of STAT3 protein expression was determined using immunoblotting, and then cell viability was measured using a CCK-8 kit. **c** Fresh whole cell lysates from MCF-7 and TamR were separated into nuclear (Lamin B1) and cytoplasmic (GAPDH) fractions. Expression level of p21 protein was determined using immunoblotting. **d** After 4 h of pre-incubation with the indicated concentration of stattic, cells were treated with tamoxifen. Fresh whole cell lysates were fractionated and immunoblotted for p21 protein expression. W: whole cell lysates; C: cytoplasmic fraction; N: nuclear fraction. Expression of GAPDH and Lamin B indicates the purity of the cytoplasmic and nuclear fractions, respectively. The cropped blots are used in the figure. The membranes were cut prior to exposure so that only the portion of gel containing the desired bands would be visualized. Each experiment was performed at least three times; representative results are shown. Data are expressed as mean ± SD and all experiments were performed in triplicate, independently. *P* values calculated using an unpaired t-test. *p < 0.05, **p < 0.01, ***p < 0.001

To assess the functional significance of STAT3 expression in TamR cells, stattic was used to inhibit STAT3 expression. Drug concentration and treatment time were determined by measuring concentration- and time-dependent cell viability and suppression of target protein in MCF-7 and TamR cells. Inhibition of STAT3 resulted in inhibition of cell proliferation by up to 20% in TamR cells compared with control or tamoxifen alone (*p* < 0.0001) (Fig. 4a). Colony formation was nearly completely inhibited by stattic in TamR cells (107% vs. 1%, *p* < 0.0001) (Fig. 4b). Annexin V-PI staining showed that inhibition of STAT3 in TamR cells increased apoptotic cell death from 18.5% (control) to 81.2% (stattic) (*p* < 0.0001) (Fig. 4c). The Expression of cleaved PARP in TamR cells was also increased by stattic compared with control or tamoxifen alone (Fig. 4d). These findings demonstrated that inhibition of STAT3 significantly inhibited cell proliferation and induced apoptosis in TamR cells, and that this was more powerful than the effect by inhibition of Src. Stattic significantly inhibited expression level of p-STAT3 protein, as well as that of p-Src and p-EGFR proteins, in TamR cells (Fig. 4d). Stattic also effectively suppressed the downstream effector molecules including cyclin D1, cyclin D3, and survivin (Fig. 4d). We examined the combined effect of stattic and dasatinib on cell proliferation in TamR cells, but there was no significant difference in cell viability between stattic alone and combined stattic and dasatinib (Fig. 5a). To verify this observation, we evaluated the effects of lacking STAT3 on recovery of tamoxifen resistance using knockdown of STAT3 by siRNA (Fig. 5b). Knockdown of STAT3 expression had no effect on sensitivity to tamoxifen in MCF-7 cells, while that enhanced sensitivity to tamoxifen in TamR cells (*p* = 0.004) (Fig. 5b).

### Inhibition of STAT3 is associated with upregulation of nuclear p21

We evaluated the mechanism that inhibition of STAT3 induced cell death in TamR cells. Considering the relevance between STAT3 signalling and cell cycle progression, we focused on the cell cycle regulator, p21(Cip1). The CDK inhibitor p21(Cip1) has a dual role in carcinogenesis, acting as an oncogenic protein or a tumour suppressor, depending on subcellular localization in the cytoplasm or nucleus. We fractionated the cytoplasmic and nuclear fractions of parental MCF-7 cells and TamR cells and found significantly decreased p21(Cip1) protein expression level in the nuclear fraction of TamR cells (Fig. 5c). We examined the expression level of p21(Cip1) in each fraction after treatment with stattic, and found that inhibition of STAT3 was associated with an increased nuclear fraction of p21(Cip1) (Fig. 5d). This effect was not observed in TamR cells treated with dasatinib (data not shown). Our data suggest that an increase in nuclear p21(Cip1) may be a key step in STAT3 inhibitor-induced cell death in TamR cells.

## Discussion

Endocrine therapy is the mainstay treatment in HR-positive breast cancer, and several mechanisms of resistance to endocrine therapy have been identified in the past decades. These include loss or mutations of ER, crosstalk between ER and other growth factor receptor signalling pathways, constitutive activation of intracellular signalling pathways such as PI3K/Akt/mTOR, alteration of cell cycle regulation, and epigenetic modification. Although various methods have been tried to overcome therapeutic resistance in the different types of cancer, only two agents, mTOR inhibitor and CDK4/6 inhibitor, are currently available in the management of endocrine resistance, and there are still unmet therapeutic needs to improve outcomes [1, 2, 13–16].

In this study, we found that the EGFR and Src-mediated STAT3 signalling pathway was significantly activated in breast cancer cells with acquired resistance to tamoxifen through gene set analysis and protein expression screening. Previous studies have reported the association between EGFR and tamoxifen resistance in ER-positive breast cancer. Massarweh et al. showed that tamoxifen-resistant MCF-7 tumors increased the expression of EGFR and HER2 and EGFR inhibitor improved the anti-tumor effect of tamoxifen [17]. Moerkens et al. reported that EGFR-driven signalling overruled the tamoxifen-mediated inhibition of estrogen-driven cell proliferation in MCF-7 cells with ectopic expression of EGFR [18]. Unexpectedly, the PI3K/Akt pathway was suppressed in TamR cells compared with control cells. Although constitutive activation of PI3K/Akt/mTOR signalling is a known resistance mechanism, opposite results have been reported in previous studies. Leung et al. reported that two hormone-resistant sublines from MCF-7 showed reduced PI3K/Akt/mTOR signalling [19]. These findings indicate that rather than a single mechanism, there are several different mechanisms related to endocrine resistance; thus, individualized treatment strategies are needed for each mechanism.

Recent studies have revealed the potential role of STATs in tumorigenesis. STAT proteins are intracellular transcription factors that constitute a family of seven members: STAT1, STAT2, STAT3, STAT4, STAT5a, STAT5b, and STAT6 [5]. STATs are activated via phosphorylation, which is mediated by Janus Kinase (JAK) associated with cytokine receptors, growth factor receptors such as EGFR, and non-receptor tyrosine kinases such as Src [5]. In particular, STAT3 and STAT5 are known to play a crucial role in carcinogenesis and drug resistance by regulating many genes related to cell proliferation and survival, including cyclin D1, cyclin D3, survivin, and c-myc [5, 20]. Recently, Jubair et al. reported 34 genes to predict the survivability of breast cancer patients using a novel network-based approach. They reported that STAT3 was one of the genes associated with survival in patients with breast cancer [21]. In the present study, activation of STAT3 was observed in TamR cells, not STAT5, and accompanied by Src-mediated phosphorylation of tyrosine 845 (Y845) in EGFR, indicating activation of a unique Src–EGFR (Y845)–STAT3 pathway in TamR cells. Sato et al. reported that Src directly phosphorylates EGFR on Y845, which is not an EGFR autophosphorylation site, and that these intracellular communications between EGFR and Src can contribute to tumorigenesis [3]. Subsequent studies have revealed that STAT3 and STAT5 are downstream effectors of the Src–EGFR (Y845) oncogenic pathway [4, 6].

Several in vitro studies have suggested that Src plays an important role in endocrine resistance. Hiscox et al. suggested that increased Src and EGFR activity increased cellular invasion and motility in breast cancer cells with tamoxifen resistance [22, 23]. Larsen et al. showed that expression and activity of Src were increased in the resistant cells, and that suppression of Src inhibited tamoxifen-resistant cell growth [24]. We also found that inhibition of Src by dasatinib inhibits cell proliferation and induces apoptosis in TamR cells, but this effect was modest. Based on our study, one reason for this limited effect is that inhibition of Src may be insufficient fully to suppress STAT3 activity, resulting in the continuous activity of downstream STAT3 target genes. Several studies have demonstrated that the effect of Src inhibitor on STAT3 activity depends on the type of tumour cells, and that inhibition of Src was not enough to suppress STAT3 activity. Hughes et al. showed that dasatinib inhibits phosphorylation of STAT3 in 4 T1 murine mammary adenocarcinoma cells, but not in MDA-MB-231 breast cancer cells [25]. Lue et al. reported that dasatinib fails to abrogate the phosphorylation of STAT3 in renal cell carcinoma cell lines [26]. Song et al. showed that dasatinib had no effect on STAT3 phosphorylation levels in most cell lines except one lung cancer cell line [27]. This may explain why dasatinib produced modest cell growth inhibition in TamR cells.

Few studies have evaluated STAT3 in endocrine resistance. Yi et al. reported that STAT3 phosphorylation is constitutively retained and upregulates anti-apoptotic signals in tamoxifen-resistant MCF-7 cells [28]. Wang et al. showed that STAT3 knockdown increases sensitivity to tamoxifen in tamoxifen-resistant breast cancer cells [29]. Bui et al. reported that tamoxifen-resistant MCF-7 cells showed constitutive STAT3 phosphorylation, and Notch inhibition decreased the level of phosphorylated STAT3 [30]. Simoes et al. showed the importance of STAT3 signalling in cancer stem-like cell-mediated resistance to endocrine therapy [31]. We found that inhibition of STAT3 by stattic effectively inhibits cell proliferation and induces apoptosis in TamR cells, and fully suppresses the downstream target molecules of STAT3. Recent advances in the oncogenic function of STAT3 indicate that STAT3 inhibitors may be a highly promising potential therapeutic cancer intervention. Although no small-molecule inhibitors of STAT3 have yet been approved to treat cancer, some early-phase clinical trials with compounds that inhibit STAT3 activity (AZD9150 or napabucasin) show anti-tumour effects in patients with advanced carcinoma [32, 33]. Accumulating clinical data that target STAT3 may provide insight into improving specificity and selecting proper patients, thus, it is expected that STAT3 inhibitors will soon be introduced into clinical practice.

p21(Cip1), a small protein belonging to the CIP/Kip family of CDKs inhibitors, was introduced as a tumour suppressor through inhibition of cell cycle progression [33]. However, recent evidence has demonstrated that p21(Cip1) has a dual role in cancer, involving tumor suppression in the nucleus and oncoprotein in the cytoplasm [34, 35]. Nuclear p21(Cip1) can induce cell cycle arrest in G1/S and G2/M transitions by inhibition of CDK4,6/cyclin D and CDK2/cyclin E [33]. Cytoplasmic p21(Cip1) has a direct anti-apoptotic function by interfering with the activity of several caspases [34, 36]. We found that cell cycle progression in G1/S was promoted in TamR cells compared with MCF-7 cells, and thus examined the expression of nuclear and cytoplasmic p21 in both cell lines. Interestingly, TamR cells have low nuclear p21 expression, consistent with their cell cycle progression and aggressive phenotype. Inhibition of STAT3 showed an increase in nuclear p21(Cip1), which is associated with cell cycle arrest and cell death. Therefore, our data suggest that induction of nuclear p21(Cip1) might contribute to a strong apoptotic effect by inhibition of STAT3.

## Conclusions

While previous studies have focused exclusively on the individual roles of Src or STAT3 in endocrine resistance, our study demonstrates a unique role of Src–EGFR (Y845)-mediated STAT3 activation and subsequent association with nuclear p21(Cip1) in endocrine resistance. Further studies are warranted to evaluate the exact role of STAT3 in p21 cellular localization. Our data suggest that inhibition of STAT3 may be a treatment option for patients with endocrine-resistant breast cancer through Src/EGFR/STAT3 activation.

## Supplementary Information

**Additional file 1: Supplementary Figure 1. a.** The protein expression levels of STAT5 and p-STAT5 were determined by immunoblotting in MCF-7 and TamR cells. Negative control: untreated HeLa cells; Positive control: interferon-alpha-treated HeLa cells. **b.** MCF-7 and TamR cells were analysed using a western blot, with the indicated antibodies, to determine the expression level of cell cycle-related molecules. The cropped blots are used in the figure. The membranes were cut prior to exposure so that only the portion of gel containing the desired bands would be visualized. GAPDH was used as the loading control.

## Data Availability

The datasets generated or analyzed during this study are accessible on reasonable request from the corresponding author.

## Abbreviations

ER: Estrogen receptor
PR: Progesterone receptor
HER2: Human epidermal growth factor receptor 2
HR: Hormone receptor
EGFR: Epidermal growth factor receptor
STAT: Signal transducer and activator of transcription protein
TamR: Tamoxifen-resistant breast cancer
FBS: Fetal bovine serum
4-OH-TAM: 4-hydroxytamoxifen
PBS: Phosphate-buffered saline
CDK: Cyclin-dependent kinase
Rb: Retinoblastoma
PARP: Poly (ADP-ribose) polymerase
GSEA: Gene set enrichment analysis
SD: Standard deviation
PI3K: Phosphoinositide 3-kinase
MAPK: Mitogen-activated protein kinase
JAK: JANUS Kinase
