# Supplementary figures and images for: Inhibition of STAT3 enhances sensitivity to tamoxifen in tamoxifen-resistant breast cancer cells
Source: BMC Cancer. 2021 Aug 18;21:931. doi: 10.1186/s12885-021-08641-7 (PMC8371881; doi:10.1186/s12885-021-08641-7)

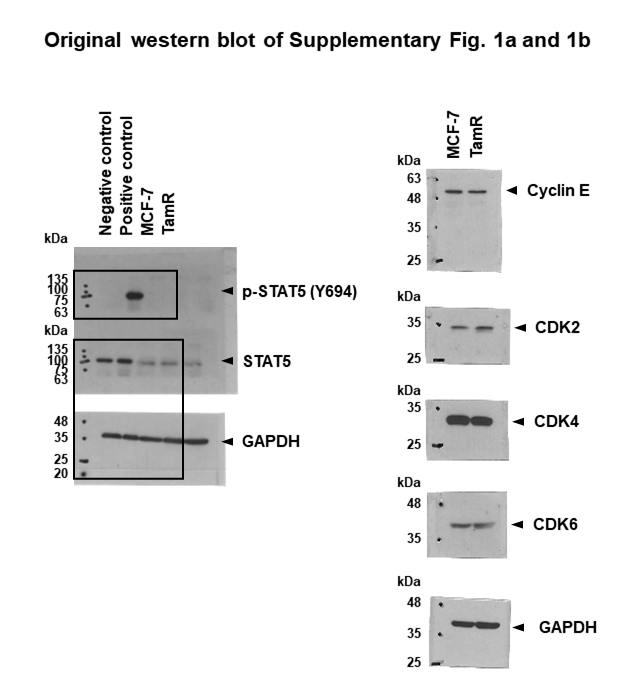

Supplement: Supplementary file 1 — Additional file 1: Supplementary Figure 1. a. The protein expression levels of STAT5 and p-STAT5 were determined by immunoblotting in MCF-7 and TamR cells. Negative control: untreated HeLa cells; Positive control: interferon-alpha-treated HeLa cells. b. MCF-7 and TamR cells were analysed using a western blot, with the indicated antibodies, to determine the expression level of cell cycle-related molecules. The cropped blots are used in the figure. The membranes were cut prior to exposure so that only the portion of gel containing the desired bands would be visualized. GAPDH was used as the loading control. [file 12885_2021_8641_MOESM1_ESM.tif]
